# Comprehensive assessment of TP53 loss of function using multiple combinatorial mutagenesis libraries

**DOI:** 10.1038/s41598-020-74892-2

**Published:** 2020-11-23

**Authors:** Vincent Carbonnier, Bernard Leroy, Shai Rosenberg, Thierry Soussi

**Affiliations:** 1 Centre de Recherche des Cordeliers, INSERM, U1138, Paris, France; 2 Sorbonne Université, UPMC Univ Paris 06, 75005 Paris, France; 3 Gaffin Center for Neuro-Oncology, Sharett Institute for Oncology, Hadassah-Hebrew University Medical Center, Jerusalem, Israel; 4 The Wohl Institute for Translational Medicine, Hadassah – Hebrew University Medical Center, Jerusalem, Israel; 5 Department of Oncology-Pathology, Karolinska Institutet, Bioclinicum J6:30, Akademiska Stråket 1, 171 64 Solna, Sweden

**Keywords:** Cancer, Computational biology and bioinformatics

## Abstract

The diagnosis of somatic and germline *TP53* mutations in human tumors or in individuals prone to various types of cancer has now reached the clinic. To increase the accuracy of the prediction of TP53 variant pathogenicity, we gathered functional data from three independent large-scale saturation mutagenesis screening studies with experimental data for more than 10,000 TP53 variants performed in different settings (yeast or mammalian) and with different readouts (transcription, growth arrest or apoptosis). Correlation analysis and multidimensional scaling showed excellent agreement between all these variables. Furthermore, we found that some missense mutations localized in *TP53* exons led to impaired *TP53* splicing as shown by an analysis of the *TP53* expression data from the cancer genome atlas. With the increasing availability of genomic, transcriptomic and proteomic data, it is essential to employ both protein and RNA prediction to accurately define variant pathogenicity.

## Introduction

The analysis of somatic and germline *TP53* mutations in human cancer is becoming clinically pertinent in numerous settings. In several hematological malignancies such as chronic lymphocytic leukemia (CLL), acute myeloid leukemia (AML), and myelodysplastic syndrome, *TP53* status is used to identify patients likely to benefit from specific treatment^1,2^. Furthermore, the early identification of germline *TP53* mutations has been shown to be highly beneficial for disease surveillance in patients with Li-Fraumeni syndrome or families with hereditary breast and ovarian cancer syndrome^3^. Today, high-throughput next generation sequencing (NGS) has ended the analysis bottleneck, but meeting quality requirements for clinical diagnostics used in personalized medicine requires the careful analysis of an exponentially-growing amount of genomic data.

One of the most unusual aspects of the *TP53* gene is the high frequency of somatic and germline missense mutations that occur in it, which is particularly unusual for a tumor suppressor gene^4^. This specific selection is believed to be linked to the antimorphic and/or neomorphic activities of the variants that transform the tumor-suppressive wild-type TP53 into a mutant oncogene.

An analysis of the most recent release of the UMD_TP53 database in the context of the TCGA showed that nearly any of the 393 residues of the TP53 protein can be found mutated in a human tumor, albeit at very different frequencies^5^. Determining the impact of all these mutations on protein functions will thus be essential. Multiple prediction methods are currently available, using information related to phylogenetic sequence conservation, amino acid physicochemical properties, functional domains and structural attributes^6^. Furthermore, several meta-tools integrating various predicting methods have been developed, such as Condel, PONP2, or REVEL^7^. More recently, machine learning has been used to develop algorithms that improve variant classification. Although many of these methods have been used for the prediction of TP53 variants, none of them have reached sufficient specificity and sensitivity for routine use.

The availability of functional data is a tremendous advantage in the analysis of TP53 variants. In 1994, we performed the first systematic analysis of 30 TP53 variants and demonstrated in that work a high heterogeneity of TP53 variant loss of function^8^. In 2003, C. Ishioka’s group released the first large-scale functional analysis of more than 2000 TP53 variants^9^. Using a convenient yeast assay, they were able to define the transcriptional activity of those variants using eight different reporter genes. Further studies showed that there was an excellent correlation between the loss of TP53 activity and the frequency of TP53 variants in human cancer^10^. That information was quickly included in a number of databases and is currently the most informative and useful parameter to infer TP53 variant pathogenicity. More recently, this functional analysis approach was extended to a larger panel of TP53 variants in two new studies, each employing different read-outs to infer TP53 variant activities^11,12^. In both of them, TP53 variants were expressed in mammalian cells and activities such as growth arrest or apoptosis were analyzed. Whether data from these three functional analyses, each using different read-outs, are in agreement for each TP53 variant has not yet been determined. In the present study, we performed an in-depth correlation analysis using these three large datasets of TP53 activities. Taking advantage of a newly devised set of pathogenic TP53 variants, we demonstrate excellent correlation between the three datasets. Furthermore, we show that cancer variants with intact protein functionality can display impaired splicing and RNA stability. This latter aspect suggests that the determination of variant pathogenicity systematically through protein function falls short of optimal and that it can be improved by systematically including information on RNA expression to increase the accuracy of predictive analysis.

## Results

### Mutability of the TP53 open reading frame in human cancer

**Figure 1:**
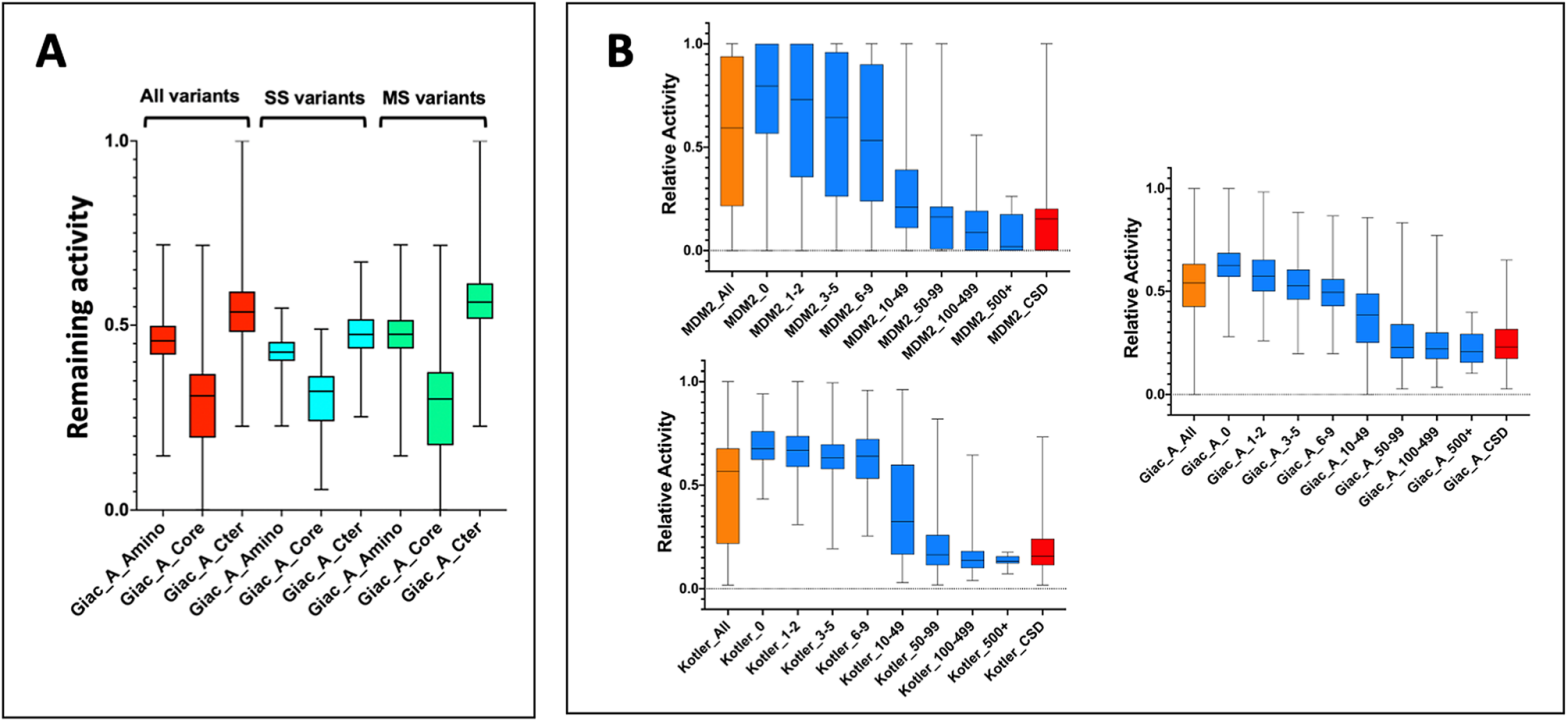
Functional analysis of TP53 variants. (**A**) TP53 variants from the core domain resulting from SNS, DNS and TNS are functionally impaired. TP53 variants were separated into 3 categories: amino (residues 1 to 99), core (residues 100 to 300) and carboxy (residues 301 to 393). Remaining activity ranging from 0 (no activity) to 1 (full activity) was determined from the normalized data of Giacomelli et al.^12^ (see "Methods"). All variants: all types of substitutions; SS: SNS variants; MS: DNS and TNS variants. (**B**) Analysis of the activity of TP53 variants according to their frequency in the UMD_TP53 Mutation Database. Boxplots display TP53 variant loss of activity from the whole database (orange plot) or from various datasets with TP53 mutants classified into eight categories according to their frequencies in the database (blue plot). CSD data are shown in red. The read-outs are identified on the x-axis with data from the three different datasets, Kato et al. (top), Kotler et al. (middle) and Giacomelli et al. (bottom). The Y axis shows variant-normalized TP53 functionality.

For the present study, we focused on missense variants as they are the most frequent modifications observed in the TP53 gene and the most difficult to predict. The *TP53* open reading frame (ORF) (CCDS11118.1) contains 1185 nucleotides, 393 significant codons and a single terminator codon. Theoretically, each codon can sustain one, two or three bases substitutions, i.e., 24,822 potential variants (Supplementary Fig. 1). In human tumors, the vast majority of TP53 (99.4%) variants result from single nucleotide substitutions (SNS) and very few from two or three substitutions in the same codon (Supplementary Fig. 1). In a previous study, we showed that TP53 variants resulting from dinucleotide (DNS) or trinucleotide substitutions (TNS) at hotspot codons 175, 248 and 273 could be highly deleterious for TP53 activity but were never observed in human cancer due to the very low probability of such events^8^. Although the first saturation mutagenesis of TP53 performed by Kato et al. included only SNS, the two recent studies performed by Kotler et al. and Giacomelli et al. included SNS, DNS and TNS in either the core domain of TP53^11^ or the entire TP53 ORF^12^. Variants in the core domain of TP53, whether they result from SNS or DNS and TNS, lead to a loss of function of the TP53 protein (Fig. 1A). The absence of DNS and TNS variants in the UMD_TP53 database (or any other cancer database) is coherent with their rarity in cancer genomes.

### Relation between TP53 loss of activity and frequency in the UMD_TP53 database

The UMD_TP53 database has been steadily updated since its creation in 1991. It is furthermore regularly and carefully curated to remove artifactual data. UMD_TP53 was the first database to integrate the functional data from Kato et al.^13,14^.

A full analysis of the 2018 release of the database was recently published in collaboration with the TCGA and will not be reiterated here. The new release of the database (100K_UMD TP53 database, October 2019) includes more than 100,000 TP53 mutations and a novel feature developed for the analysis of TP53 variants, the cancer shared dataset (CSD), which comprises only variants highly likely to be pathogenic^15^ (see "Methods"). The CSD includes 258 TP53 SNS common to four different large independent sequencing datasets (see "Methods"). In addition to its hotspot variants found highly frequently in various types of cancer, the CSD also includes less common variants that would have been missed in a selection based solely on mutation frequency. The 100K_UMD TP53 database now includes the data of the two recent saturation mutagenesis studies performed by Kotler et al. and Giacomelli et al.^11,12^. The database thus comprises 12 different read-outs for each TP53 variant, with 8, 3 and 1 read-outs resulting respectively from the works of Kato et al., Giacomelli et al., and Kotler et al. (see "Methods").

Analysis of the loss of activity of TP53 variants in relation to their frequency in the database is shown in Fig. 1B, Supplementary Fig. 2. First, for the 12 different readouts, we confirmed a clear correlation between the TP53 variants’ losses of activity and their frequency in human tumors. Second, TP53 single nucleotide variants that are never found in human cancer displayed a strong propensity for keeping their wild-type activity and had profiles similar to those of uncommon cancer variants. Third, the profiles of TP53 variants included in the CSD were the same as those of frequent TP53 variants, despite different frequency distributions in human tumors (Fig. 1B and Supplementary Fig. 2.).

**Figure 2:**
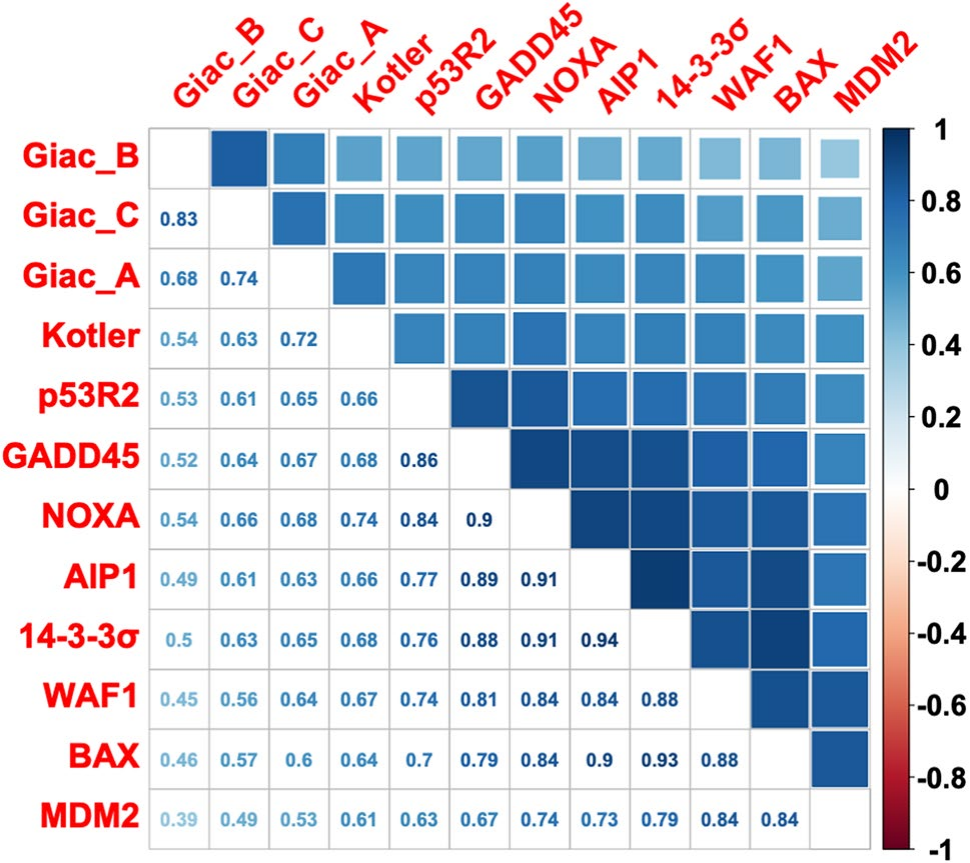
Correlation matrix for the three large-scale (12 read-outs) analyses of TP53 activity. Correlation R values are shown on the left part of the panel. Positive correlations are displayed in blue and negative correlations in red. Color intensity and the size of the square are proportional to the correlation coefficients. On the right side of the correlogram, the legend color shows the correlation coefficients and the corresponding colors. All correlations are highly significant (p < 0.001) (see supplementary Fig. 3 for a detailed view of the statistics).

### Correlation analysis for the three large-scale mutagenesis analyses of TP53

**Figure 3:**
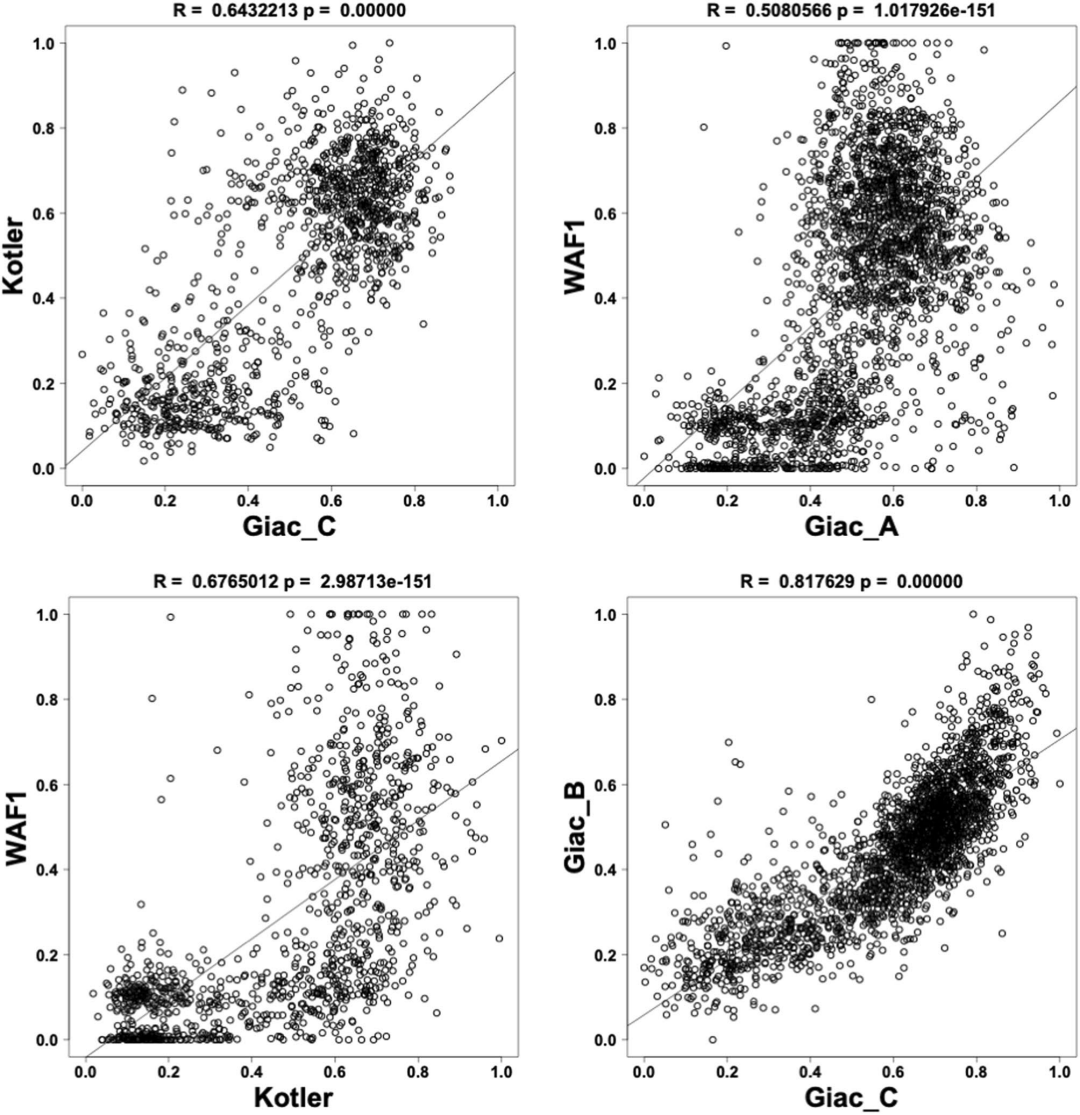
Binary correlation analysis between the various read outs of TP53 loss of activity. Overall Pearson R correlation coefficients and p values are shown at the top of each figure. Data for other read-outs are shown in Supplementary Fig. 3.

**Figure 4:**
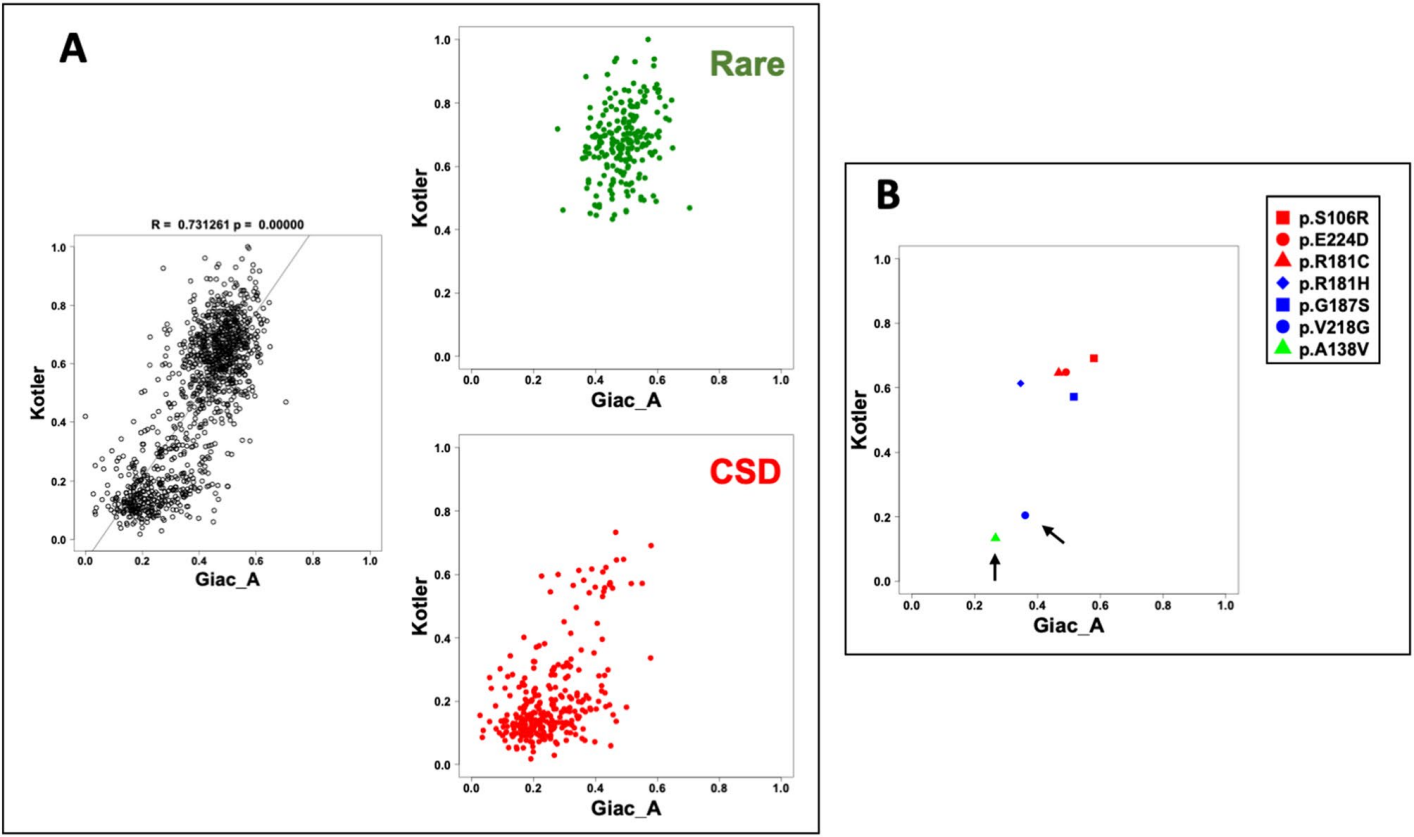
(**A**) TP53 variants can be divided into two classes according to their loss of activity. The binary comparison between two studies (Kotler and Giac_A) has been split into two panels, with variants found in the CSD in the lower right panel (red) and rare TP53 variants (variants found once in or absent from the database) in the upper right panel (green). Data for other read-outs are shown in Supplementary Fig. 3. (**B**) Position of the 7 CSD outlier variants in different binary comparison. Two variants, p.A138V and p.V218G (shown by black arrows), display no loss of activity in the yeast assay (top figures) but are inactive using the various mammalian readouts (bottom figures). Other variants associated with splice defects are also displayed in the figure. Data for other read-outs are shown in Supplementary Fig. 5.

**Figure 5:**
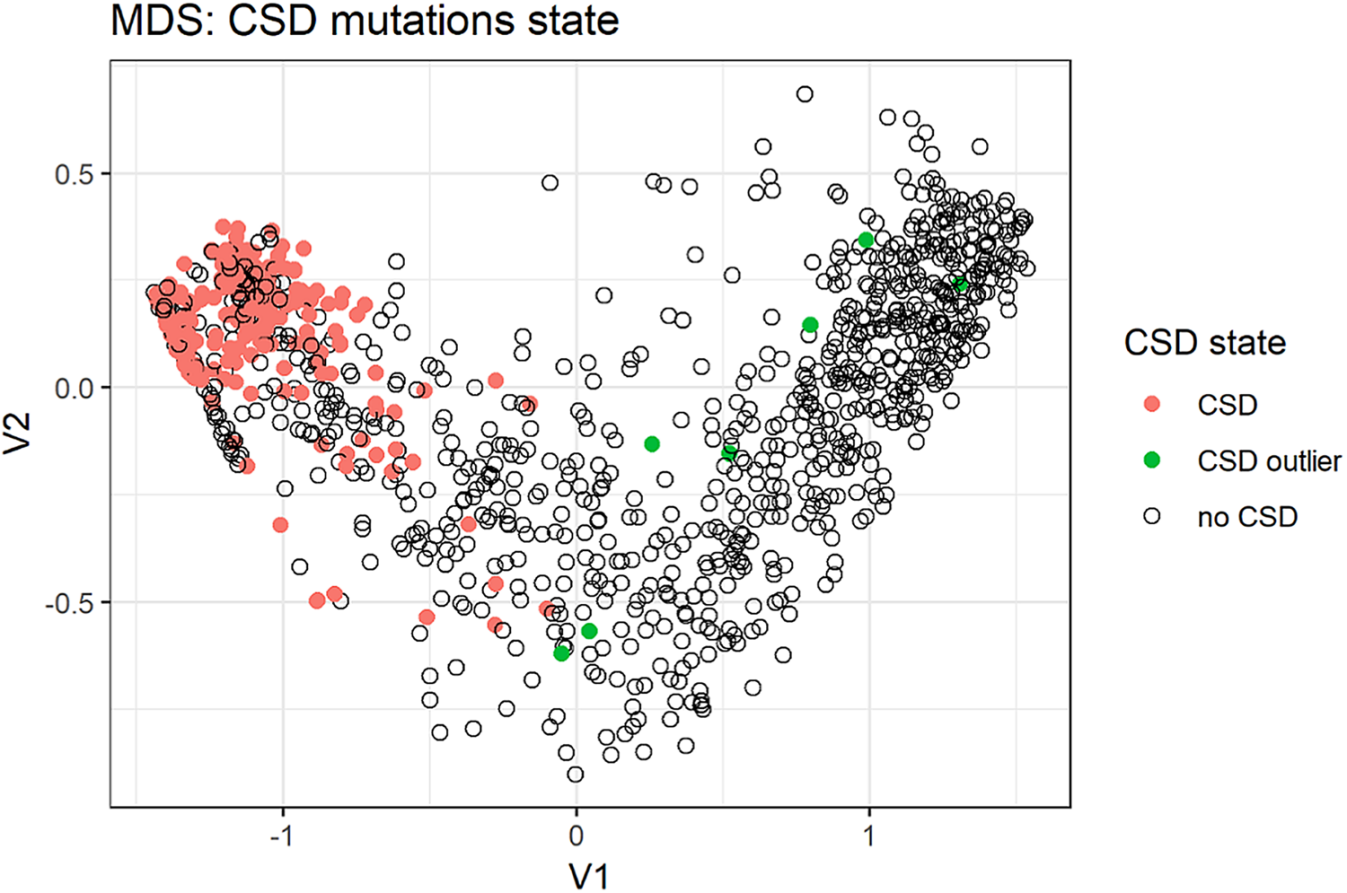
Multidimensional scaling (MDS) for reduction of the 12 activity measures into bidimensional space. CSD, CSD outliers and no CSD mutations are colored red, green and black respectively.

The analysis shown in Fig. 1B revealed a clear relation between TP53 variant frequency and TP53 loss of activity for each functional study (each using a different read-out for TP53 function). However, it did not provide a comparison of each TP53 variant among the three studies; indeed, to date, such an analysis has never been done. Therefore, we performed a correlation analysis using the 12 read-outs available for TP53 loss of function (Figs. 2 and 3, and Supplementary Fig. 3). A correlation matrix for the three studies showed a highly significant positive correlation between them (Fig. 2). Data from the two studies performed in mammalian cells (Kotler et al. and Giacomelli et al.) showed strong correlation, with Pearson R (R) values between 0.52 and 0.72 (Fig. 2) and a highly significant *p* value (*p* < 0.001 for all binary comparison, Supplementary Fig. 3). Depending on the promoter used to report TP53 loss of activity, the correlation between the yeast-derived data from Kato et al. and those from Kotler et al. and Giacomelli et al. remained significant but with more heterogeneous R values. Of note: among the data of the eight promoters tested by Kato et al., those of the NOXA promoter, involved in apoptosis, systematically showed the best correlations with Kotler et al. (R = 0.74) and Giacomelli et al. (R = 0.54, 0.66 and 0.68 respectively for the three readouts). In binary comparisons, data tended to be distributed in two groups (Fig. 3 and Supplementary Fig. 3). To clarify the presentation, variants markedly present in the CSD datasets were colored red and those found infrequently in the database green (Fig. 4A, Supplementary Fig. 3, Supplementary Video 1). Most CSD variants clustered in the lower left corner of the graph, confirming minimal remaining activity. In contrast, rare variants were located preferentially in the upper right part of the graph, indicating functional proteins. This specific pattern, observed in all binary comparisons using the 12 different read-outs, confirms that these functional readouts are robust to rank TP53 cancer associated variants (Fig. 4A, Supplementary Fig. 3). Neverthless, there were several outlier variants included in the CSD that displayed near wild-type activity (Fig. 4A, Supplementary Fig. 3). To get a global picture of the relations between the activities of all 12 readouts, a multidimensional scaling (MDS) analysis was performed (Fig. 5, Supplementary Fig. 4). The MDS plot clearly revealed two distinct groupings, one with the pathogenic mutations from the CSD and a second with the rare mutations. To define and explore the outlier mutations, we measured the coordinates of the center of the CSD mutations on the bidimensional graph of the MDS, and thereafter the Euclidean distance of each CSD mutation from that center.

We selected the 2.5% of mutations with the largest Euclidean distances. The seven CSD variants therein were defined as outliers with minimal loss of activity (Fig. 4B, Table 1, Supplementary Fig. 5). One of them, p.A138V, described in 126 tumors in the UMD, displayed complete activity in the study performed in yeast but complete inactivity in both studies performed in mammalian cells. This variant has been shown to be thermosensitive with wild-type activity at 30 °C and inactivity at 37 °C^16^. Because the yeast assay was performed at 30 °C, it is likely that the loss of function for this pathogenic variant was misidentified (Fig. 4B Supplementary Fig. 5). Similarly, CSD variant p.V218G, described in 35 tumors in the UMD, was fully active in yeast but inactive in mammalian cells. Although this variant is not known to be thermosensitive, it is likely that the yeast assay, based on transcription, did not fully reflect losses of function. Furthermore, several variants that retain DNA binding activity have been shown to be devoid of biological activities such as growth arrest or apoptosis^17^.

**Table 1:** Alternative loss of function of the 7 CSD TP53 variants.

TP53 variant^1^	Activity^2^			Occurrence in UMD^3^	Position in the *TP53* gene	Potential loss of function
	Score according to Kato et al.^4^	Score according to Kotler et al.^5^	Score according to Giacomelli et al.^6^			
p.E224D	0.91	0.65	0.56	48	Last Base Exon	Splice variant
p.V218G	0.86	0.20	0.20	35	Exon	Differential loss of activity in yeast and mammalian cells
p.G187S	0.72	0.57	0.55	34	Exon	Splice variant
p.A138V	0.67	0.13	0.35	126	Exon	Thermosensitive; differential loss of activity in yeast and mammalian cells
p.S106R	0.51	0.69	0.63	39	Exon	RNA destabilization; possible splicing defect
p.R181H	0.44	0.61	0.50	77	Exon	Position 181 is known to impair TP53 for specific functions^7^
p.R181C	0.40	0.65	0.58	103	Exon	

^1^The sequence nomenclature used for TP53 variants in this work is in accordance with the Human Genome Variation Society's guidelines using the NM_000546.5 transcript sequence and the full-length protein NP_000537.3.

^2^TP53 functional data were scaled in the range of 0 to 1 with 0 corresponding to the lowest activity of TP53 variants.

^3^Number of tumors expressing each TP53 variant included in the TP53_UMD.

^4^Data from the transcriptional activity performed in yeast using 8 different p53 response elements (mean of the activities for the 8 readouts) from the work of Kato et al.^9^.

^5^Score from the growth arrest assay in H1299 cells performed by Kotler et al.^11^.

^6^Mean score from the three assays performed by Gacomelli et al.^12^ (see "Methods" for a full description of the assays).

^7^Both in vitro studies and mouse models indicate that these variants are functionally defective^21,32^.

**Figure 6:**
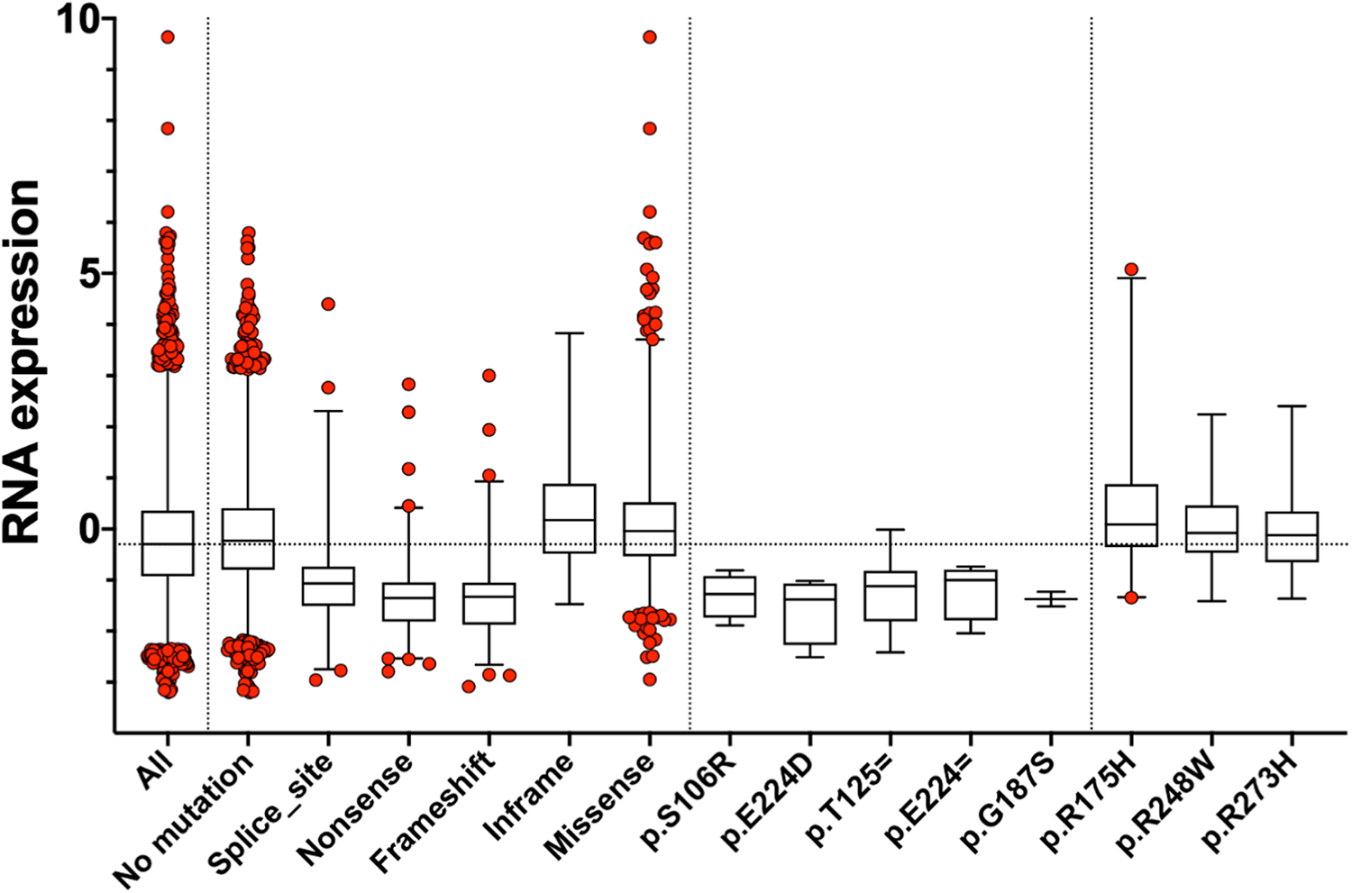
TP53 RNA expression differs in tumors depending on TP53 mutation status. Box-and-whisker plots show the interquartile ranges (boxes), median values (horizontal lines inside the boxes), and full-range distributions (whisker lines) for TP53 RNA content according to the type of TP53 mutation. RNA expression and TP53 mutational status values for multiple tumor types were extracted from cBioPortal (see "Methods").

Two other outlier CSD variants, p.G187S and p.E224D, were located at the two extremities of exon 5. Mutations at penultimate codons have been shown to be able to affect splicing^18^. In the *TP53* gene, it is well established that synonymous mutations at codons 224 and 125, two penultimate positions in exons 4 and 6, impair TP53 splicing^19^. The exclusion of specific exons from a transcript can cause a frameshift by creating a premature stop codon if the exons flanking the skipped exon are not in the same reading frame^20^. Translation of the truncated protein should induce the nonsense-mediated decay (NMD) pathway and result in the degradation of the transcript, thus preventing protein production. To gain more insights into the possible defect in RNA splicing, we used TCGA data as they include both genomic and transcriptomic information from matched samples. RNA data from 10,000 tumors were analyzed for TP53 RNA content (Fig. 6) (see "Methods"). Tumors carrying wild-type TP53 and those expressing missense TP53 variants showed similar TP53 RNA expression. In contrast, TP53 RNA expression was significantly lower in tumors expressing variants leading to the potential expression of a premature TP53 protein, thus confirming an NMD phenomenon. In a comparable manner and similarly to nonsense variants, tumors with variants in canonical splice site sequences (intronic variants at positions + 1; + 2 or −1; −2) displayed a significant decrease in TP53 (Fig. 6). Notably, no decrease of TP53 RNA was observed in tumors expressing in-frame TP53 variants, suggesting that these proteins are fully translated and no NMD is induced. TP53 RNA expression was analyzed for several individual mutants included in the study. The CSD outlier variants p.G187S and p.E224D were both associated with very low TP53 RNA contents, resembling what has been observed for splicing variants. Similar results were found for the synonymous variants p.E224 = and p.T125 = , which have been shown to be defective for TP53 splicing (Fig. 6)^19^. Missense variants at hotspot positions were not associated with decreased expression of TP53 RNA. Therefore, for these two variants located in the vicinity of an intron (p.G187S and p.E224D), the consequences of mutation manifest at the level of RNA and not that of the protein. For a third outlier variant, p.S106R, there was a sufficient number of samples to examine TP53 expression and a decrease of TP53 RNA similar to those found for splice or nonsense mutations was observed (Fig. 6). This variant is located inside exon 4, twenty codons before the exon–intron junction. Exonic sequences such as exon splice enhancers (ESE) or exon splice silencers (ESS) are known to regulate mRNA splicing and can be targeted by pathogenic mutations. We used the Human Splicing Finder (HSF) system to identify and predict the effects of mutations on splicing motifs. This included the acceptor and donor splice sites, the branch point, and auxiliary sequences known to either enhance or repress splicing. The region around codon 106 was shown to contain a SF2/ASF motif that recruits the serine/arginine-rich splicing factor 1 (SRSF1) involved in pre-mRNA splicing (Supplementary Fig. 6). The C > G mutation associated with p.S106R was strongly predicted to inactivate this site and therefore to impair TP53 splicing (Supplementary Fig. 6). Two other CSD outlier TP53 variants, p.R181H and p.R181C, were located at the same position. Recent structural studies have shown that the TP53 molecules within the tetramer, which assembles as a dimer of dimers, do not only interact through their oligomerization domains but also tightly and specifically through their DNA-binding domains including H1 helix residues 180 and 181, which have been shown to be essential for dimer stability^21,22^. Modifications at codon 181 do not fully abolish TP53 function. These variants have differential losses of function depending on the TP53 target genes. They were shown to be able to transactivate p21CDKN1A or mdm2 at levels similar to wild-type TP53 but unable to transactivate genes associated with apoptosis, such as NOXA or p53AIP1 and induce apoptosis^21,22^. Although p.R181H and p.R181C showed only partial losses of activity they should nonetheless be considered as pathogenic.

## Discussion

Advances in and the application of massive parallel sequencing have revolutionized molecular diagnostics in cancer and provided an immense quantity of information on human genetic variations^23^. Interpreting these latter, whether somatic or constitutional, has provided important insights into the genetic basis of many types of cancer, and opened new promising vistas for preventive, diagnostic, and therapeutic strategies. This vast body of genetic data has also enabled the development of successful predictive tools that integrate genetic, molecular, evolutionary, and/or structural information^7^. These predictive tools have been applied to TP53 with variable success and heterogeneous results^24,25^. The clinical relevance of TP53 diagnostics is nonetheless growing for both somatic and germline mutations^26^. The TP53 gene is the most frequently mutated gene in human cancer but it is also the gene that sustains the largest diversity of single nucleotide variants, with more than 3000 different missense variants identified so far. Although hotspot variants have been shown to be truly pathogenic, the situation is more ambiguous for rare variants. The UMD database includes 4500 TP53 missense variants that have been described only once or twice and whether they are true pathogenic variants, rare passenger mutations or sequencing artifacts is currently unknown. The greatest advantage for TP53 is that the read-out of its functions can be easily monitored. In 2003, C. Ishioka’s group published a seminal paper wherein they described the first large-scale analysis of TP53 using a transactivation assay developed in yeast^9^. Their functional data, unique for a cancer gene, have been widely used to increase the prediction of TP53 variants^10^. Nevertheless, multiple studies have shown that the relation between the transcriptional activity of TP53 and the effects on biological functions, such as growth arrest or apoptosis, is not straightforward^27^. The two recent large-scale analyses performed in mammalian cells have greatly advanced the identification of TP53 variants that sustain a loss of function^11,12^.

For the present analysis, we developed upon the CSD, a specific TP53 benchmark dataset that includes only highly likely pathogenic variants^15^. As variant recurrence is one of the strongest indicators of pathogenicity, using four independent sources of TP53 variants, each deploying a different analysis methodology, should negate methodological issues for the specific selection of pathogenic variants.

We found excellent correlation between the three saturation studies used as a base for the present study, i.e., those of Kato et al., Kotler et al., and Giacomelli et al. We showed that the yeast assay using the NOXA promoter was highly correlated with the two studies performed in mammalian cells, thus confirming the importance of this gene in the tumor suppression function of TP53^28^. Furthermore, our analysis showed that the vast majority of the variants in the CSD sustain a loss of function in the TP53 protein. The inclusion of data derived from mammalian cells increased the accuracy of the analysis with the identification of leaky pathogenic variants that were not identified in the yeast assay. In addition, several pathogenic variants found at high frequency in the various databases did not display any prominent losses of function.

Of course, gene mutations can impair mRNA, and RNA splicing and/or stability can be damaged by multiple synonymous or nonsynonymous mutations located in exons or introns^19,29^. Several motifs localized in exons, such as ESEs or ESSs, are known to modulate gene splicing. Most computational predictive algorithms rely on the properties of the protein, and functional assays force protein expression using unspliced cDNA constructs. It is therefore possible that some TP53 loss of function is due to RNA expression and not a potential inactivation of protein.

Using RNA data expression from tumors with matched TP53 status, we showed that some CSD TP53 variants without obvious loss of protein activity are in fact spliced variants associated with a loss of TP53 RNA expression.

The number of variants altering TP53 RNA is likely underestimated; although functional assays may report inactivity for the protein, it is possible that this latter was never expressed due to the lack of RNA expression.

Increasing the number of tumors with matched genomic and transcriptomic data will be the only way to improve the detection of this type of variant and define more accurately whether the ultimate target of the mutation is the mRNA or the protein. This will be essential for clinical studies targeting the TP53 protein as TP53 null tumors, whether at the DNA or RNA level, will not benefit from them.

Benchmark variants are required for developing, optimizing and assessing the performance of sequencing and bioinformatics methods. For TP53, benign variants are generally identified in ClinVar, dbSNP or gnomAD whereas their pathogenic counterparts usually emanate from cancer mutation databases. Unfortunately, both sets of data are generally not accurate. We have previously shown that gnomAD is heavily contaminated by pathogenic TP53 variants due to the high frequency of de novo mutations in the human population^15^. Similarly, as shown in previous studies, cancer mutation databases can include passenger mutations, unidentified low frequency SNP and artifactual data^30,31^. The use of the cancer shared dataset (CSD) in the present study alleviated some of these problems and did not bias the choice of pathogenic variants toward hotspot variants. Moreover, this approach can capture pathogenic TP53 variants that would be missed by functional analysis. The robustness of the CSD and the availability of multiple independent cancer datasets such as TCGA, MSKSCC, ICGC or the Locus-Specific Database (LSDB) could be easily mined to define multiple, gene-specific CSDs. Such resources would contribute to pinpointing rare pathogenic variants and also curating fast-growing population databases such as gnomAD that are essential for genetic analyses.

## Methods

### Database and datasets used for the analysis

The most recent issue of the UMD database (100K_UMD TP53 database) was released in November 2019 with 125,130 TP53 mutations retrieved from the literature. Of them, 40,624 were identified in studies using conventional Sanger sequencing and 75,339 in studies using NGS. Variants from publications using other methodologies or combining conventional sequencing and NGS were not used in the present study.

### Functional data used in the present study

Functional data from the works of Kato et al.^9^, Kotler et al.^11^, and Giacomelli et al.^12^ were used for the present study.

Transcriptional activity from the work of Kato et al. in yeast transformants containing a p53 cDNA and a green fluorescent protein reporter plasmid was assessed with eight different promoters for 2315 TP53 variants. These yeast data are currently the most widely used criteria to define the pathogenicity of TP53 variants^10^.

Kotler et al. generated a library of TP53 variants from the core domain of TP53 (residues 100 to 300) that were analyzed using a range of assays both in mammalian cell lines and in mice. Only data obtained from the over-expression of 9833 TP53 variants in H1299 cells was used in the present study as only a subset of their TP53 variants was used for other read-outs. Data from TP53 variants with two or more mutations in different codons were discarded as were nonsense and frameshift variants to focus our analysis on missense variants with one, two or three substitutions in a single codon.

Data from Giacomelli et al. included 7469 TP53 variants observed across the whole TP53 protein. The present analysis used their three different read-outs: TP53 activity in (i) wild-type A549 cells treated with nutlin was defined as the Giac_A read-out; (ii) TP53-null cells treated with nutlin as the Giac_B read-out; and (iii) TP53-null cells treated with etoposide as the Giac_C read-out. Similarly to the Kotler et al. data, only missense variants involving one, two or three substitutions in a single codon were selected.

For the three studies, TP53 functional data were normalized to a range of 0 to 1 with 0 indicating the lowest activity of TP53 variants.

### The cancer shared dataset (CSD)

The CSD concept was developed to optimize the representativeness of pathogenic variants in a dataset^15^. Previously, training sets used for defining TP53 pathogenicity were based on either the whole set of mutations found in various databases or a selection of the most frequent TP53 variants with an arbitrary cut off. Both methods showed bias: The first one included the potential passenger and artifactual variants that plague the various cancer databases and the second did not account for infrequent pathogenic variants resulting from rare genetic events such as T > A transversions. To circumvent these issues, we developed a novel strategy involving the extraction of TP53 mutation data from four non-overlapping databases: (i) TP53 variants from the UMD database that were harvested from studies using exclusively conventional Sanger sequencing for diagnostics; (ii and iii) data from the TCGA and MSKCC studies and downloaded from cBioPortal (https://www.cbioportal.org/, October 2019); and (iv) data from the ICGC portal and downloaded from the ICGC website (https://dcc.icgc.org/, data release 26 December 2017). Only missense variants found at least once in each dataset were included in the CSD. Compared to the CSD described in our previous study, we added another parameter in this selection, i.e., the choice of tumors carrying only a single TP53 mutation. That additional parameter removed the passenger mutations frequently associated with hotspot variants. The CSD used for the present study thus included a core of 258 recurrent missense TP53 variants found at least once in each database. As the four datasets were derived from independent studies using different patients and different methodologies, it was highly likely that the 258 shared variants were true recurrent pathogenic variants. This dataset was validated in a previously-published report^15^. It includes both hotspot and less frequent variants and is more representative of the heterogeneous frequency of TP53 mutation in human cancer.

### In silico analysis of TP53 splice variants

The Human Splicing Finder (HSF; https://www.umd.be/HSF/) system combines 12 different algorithms to identify and predict the effects of mutations on splicing motifs including the acceptor and donor splice sites, the branch point, and auxiliary sequences known to either enhance or repress splicing: exonic splicing enhancers (ESE) and exonic splicing silencers (ESS).

These algorithms are based on position weight matrices, the maximum entropy principle or a motif comparison method. For each of them, we defined a consensus value threshold and a score variation threshold, based on literature datasets.

### Multidimensional scaling (MDS)

We used MDS to reduce the 12 functional scores into two dimensions. Euclidean distance was calculated between every possible pair of mutations and thereafter MDS was performed to optimally locate the mutations on a bidimensional graph. The coordinates of the center of the CSD mutations were calculated as the average of the coordinates of the CSD mutations calculated separately for each of the two axes.

### RNA expression analysis

Data for TP53 RNA expression was available from 31 non-overlapping tumor studies available in cBioPortal (https://www.cbioportal.org/). TP53 status was available for all concerned tumors. Only RNA data normalized by Z score transformation was used for the analysis.

## Supplementary information

Supplementary Information 1.

Supplementary Video 1.
