# Supplementary material for: Comprehensive assessment of TP53 loss of function using multiple combinatorial mutagenesis libraries
Source: Sci Rep. 2020 Nov 23;10:20368. doi: 10.1038/s41598-020-74892-2 (PMC7683535; doi:10.1038/s41598-020-74892-2)
Supplement: Supplementary file 1 — Supplementary Information 1. [file 41598_2020_74892_MOESM1_ESM.pdf]

Comprehensive assessment of TP53 loss of function using multiple combinatorial mutagenesis libraries

Vincent Carbonnier[1], Bernard Leroy[2], Shai Rosenberg[3,4], and Thierry Soussi[1,2,5]

[1] INSERM, U1138, Centre de Recherche des Cordeliers, Paris, France
[2] Sorbonne Université, UPMC Univ Paris 06, F- 75005 Paris, France
[3] Gaffin Center for Neuro-Oncology, Sharett Institute for Oncology, Hadassah-Hebrew University Medical Center, Jerusalem, Israel
[4] The Wohl Institute for Translational Medicine, Hadassah-Hebrew University Medical Center, Jerusalem, Israel
[5] Karolinska Institutet, Department of Oncology-Pathology, Bioclinicum J6:30, Akademiska Stråket 1, 171 64, Solna, Sweden

Correspondence: thierry.soussi@sorbonne-universite.fr

Supplementary figures

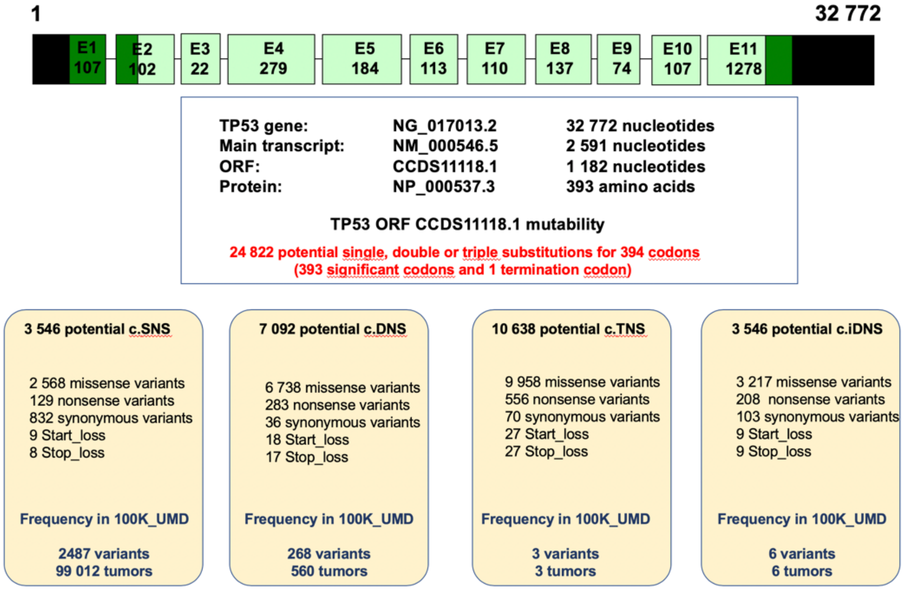

Supplementary Figure 1. Mutability of the TP53 gene. Four types of substitution can occur in a single codon ; cSNS (single nucleotide substitution at position 1, 2 or 3); cDNS (double substitution at two adjacent nucleotides, 1 and 2 or 2 and 3); c.TNS (triple nucleotide substitution at the 3 positions) and c.iDNS (interrupted double nucleotide substitution with a two substitutions at positions 1 and 3, without any change at position 2). Analysis of the 100 K UMD_TP53 database shows that more than 99% of nucleotide substitutions found in human cancer is cSNS. Other double and triple substitutions are extremely rare. Most c.DNS observed in the TP53 gene are found in skin cancer and involve two adjacent nucleotides in a dipyrimidine sites known to be targeted by UV.

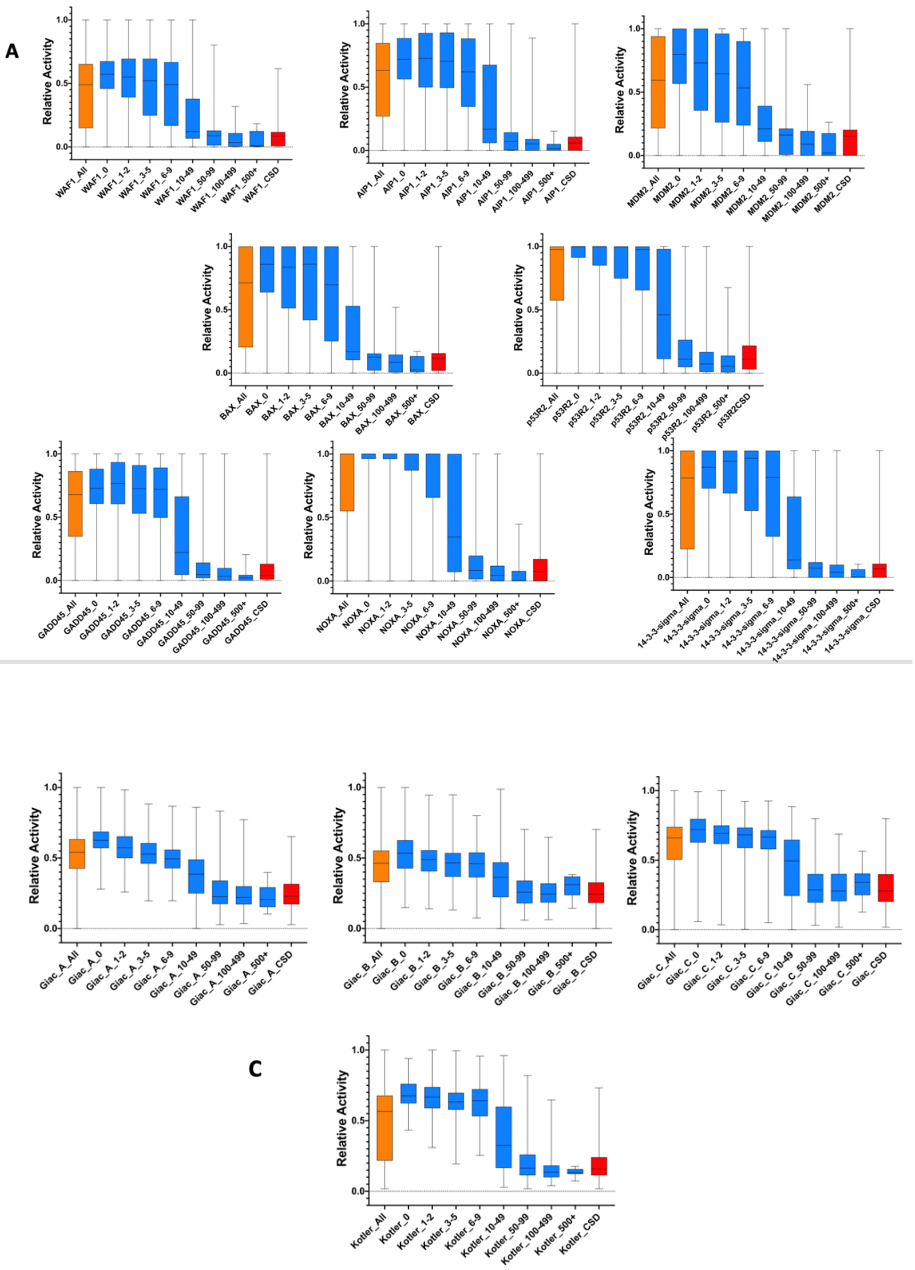

Supplementary Figure 2: analysis of TP53 variants activity according to their frequency in the UMD_TP53 Mutation Database. Boxplot plots display TP53 variant loss of activity from the whole database (orange plot) or from various datasets with TP53 mutants classified into 8 categories according to their frequencies in the database (blue plot). CSD data are shown in red. Identity of the read-out is shown in the x-axis with data from the three different datasets, Kato et al. (Top), Kotler et al. (Middle) and Giacomelli et al. (bottom). The Y axis shows variant normalized TP53 activity from 0 (no activity) to 1 (full activity).

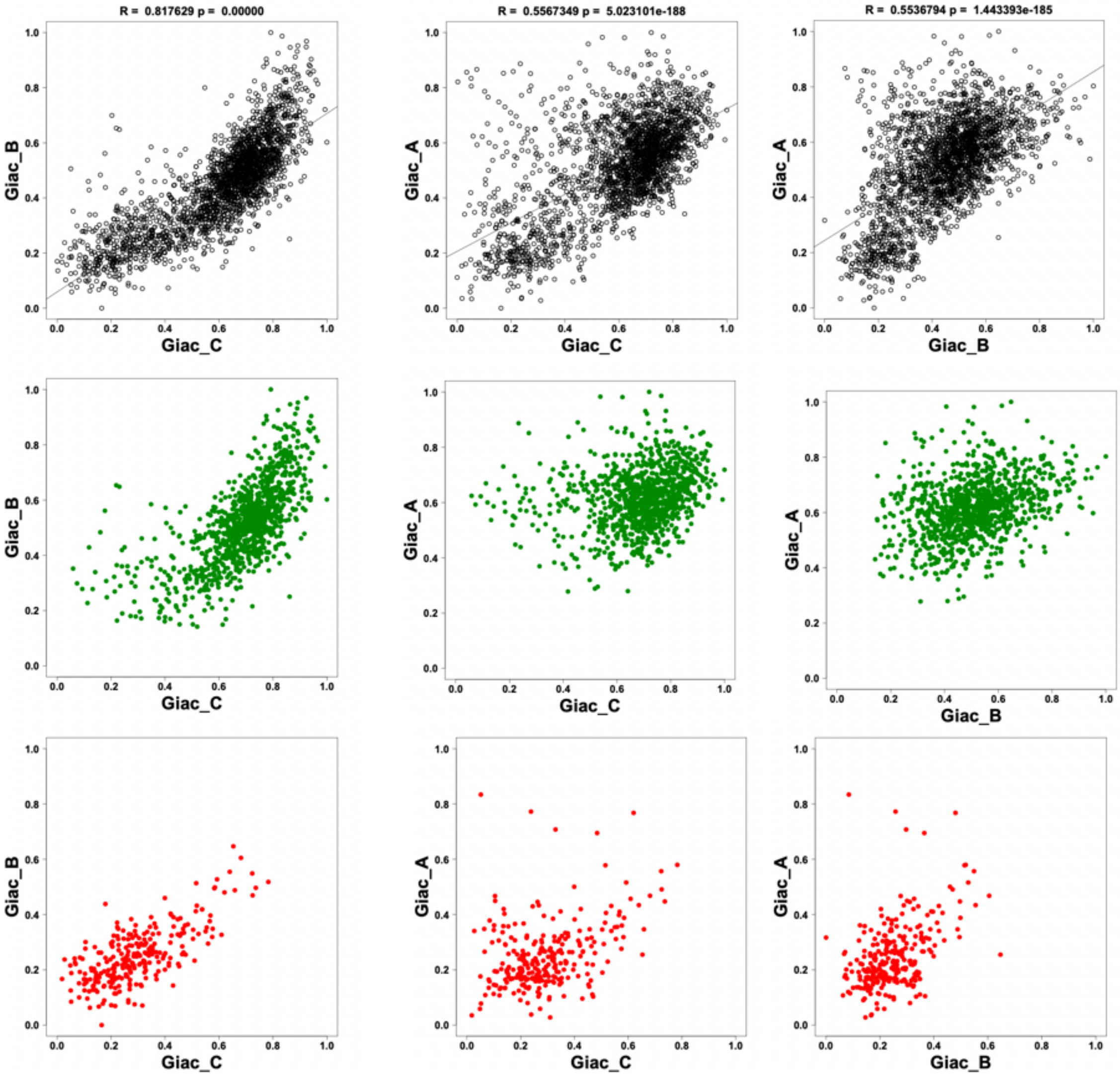

Supplementary Figure 3; panel 3A

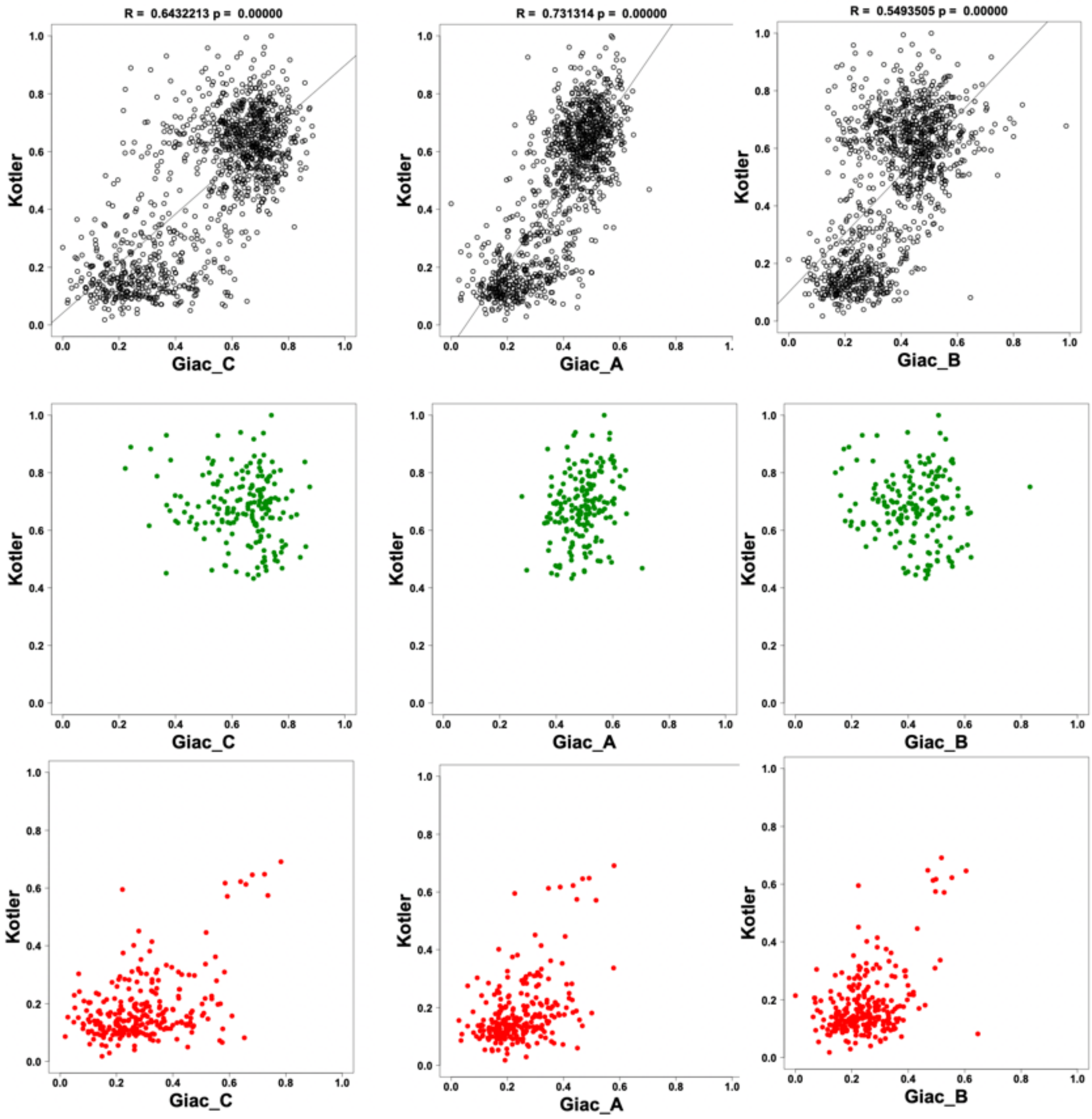

Supplementary Figure 3; panel 3A

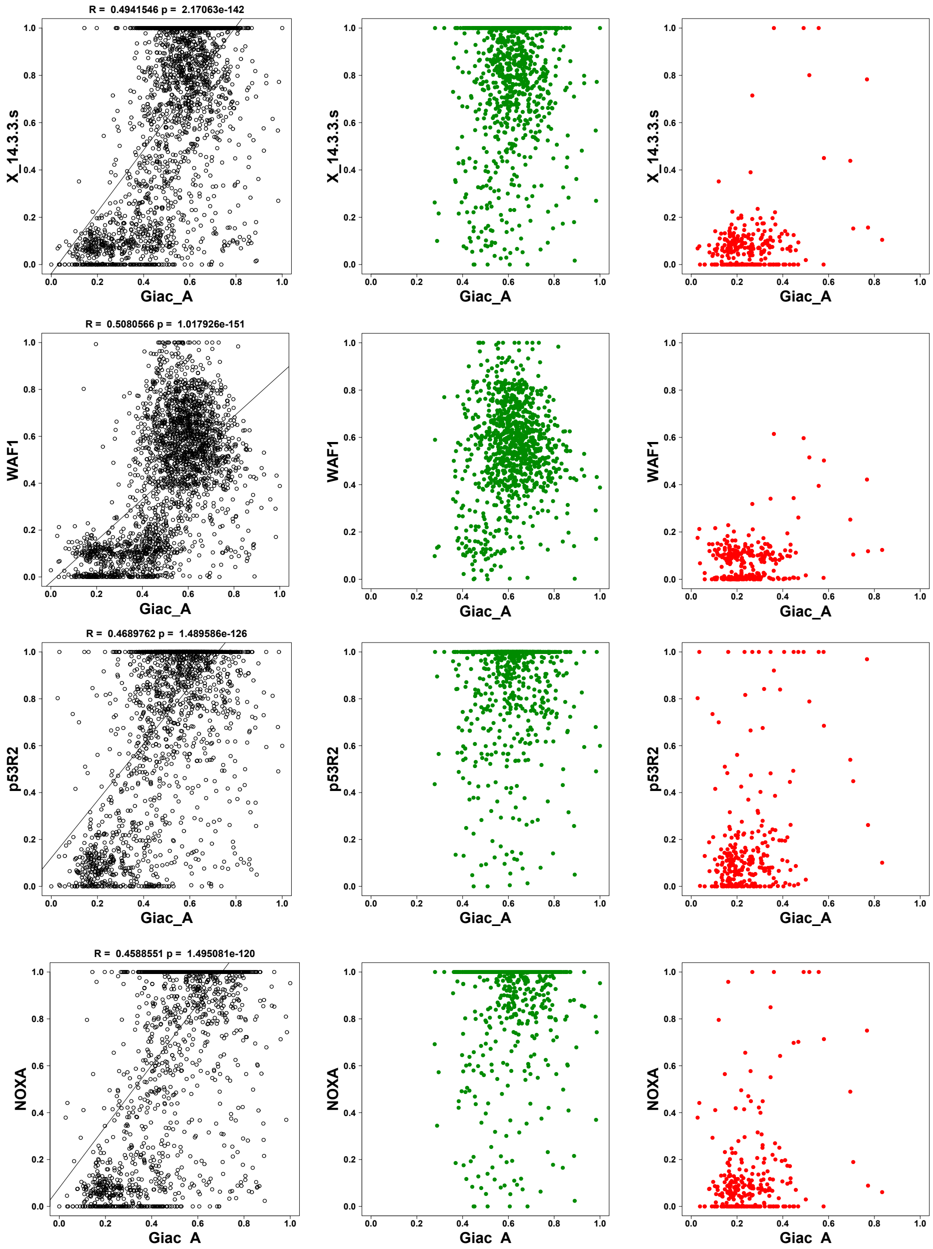

Supplementary Figure 3; panel 3B

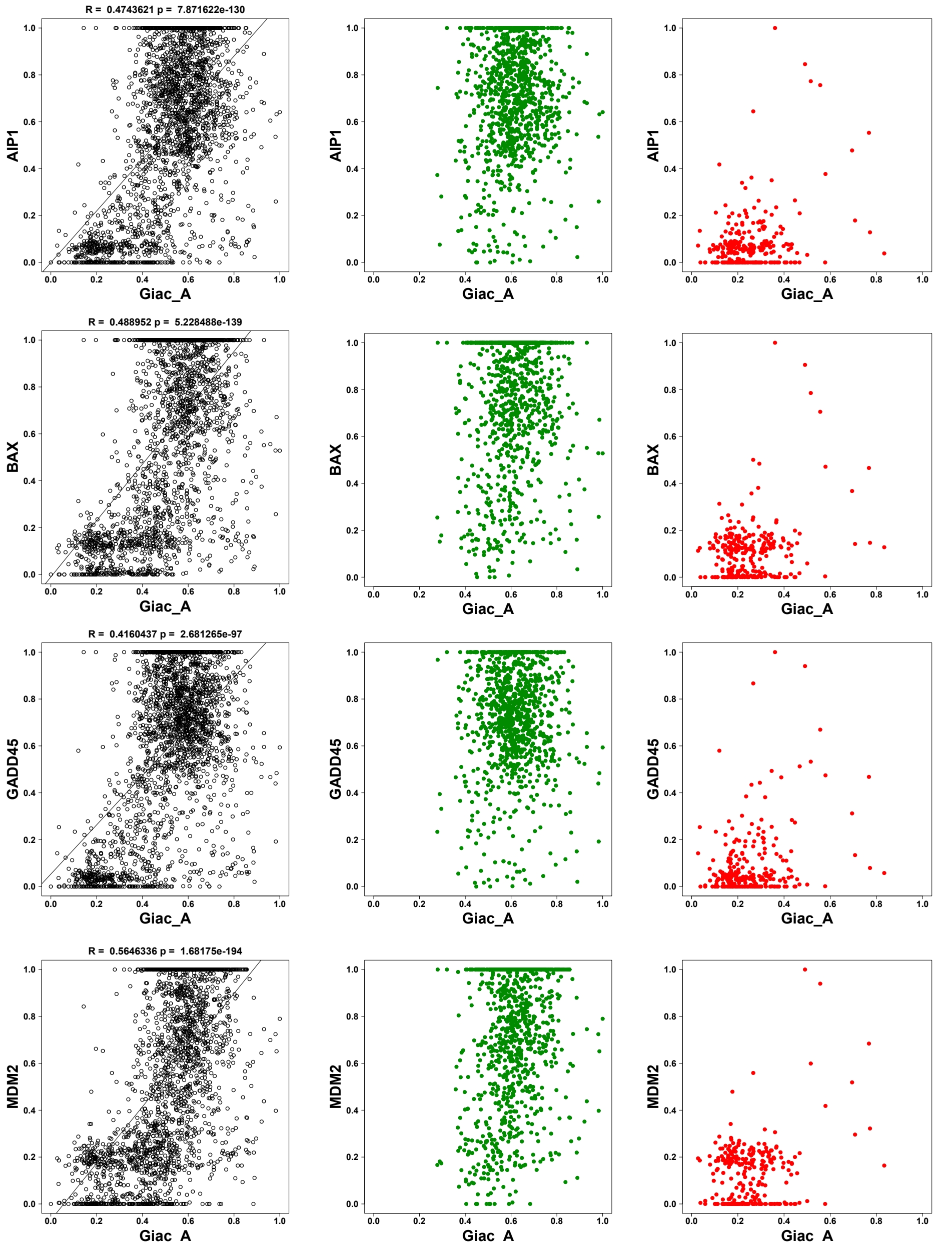

Supplementary Figure 3; panel 3B

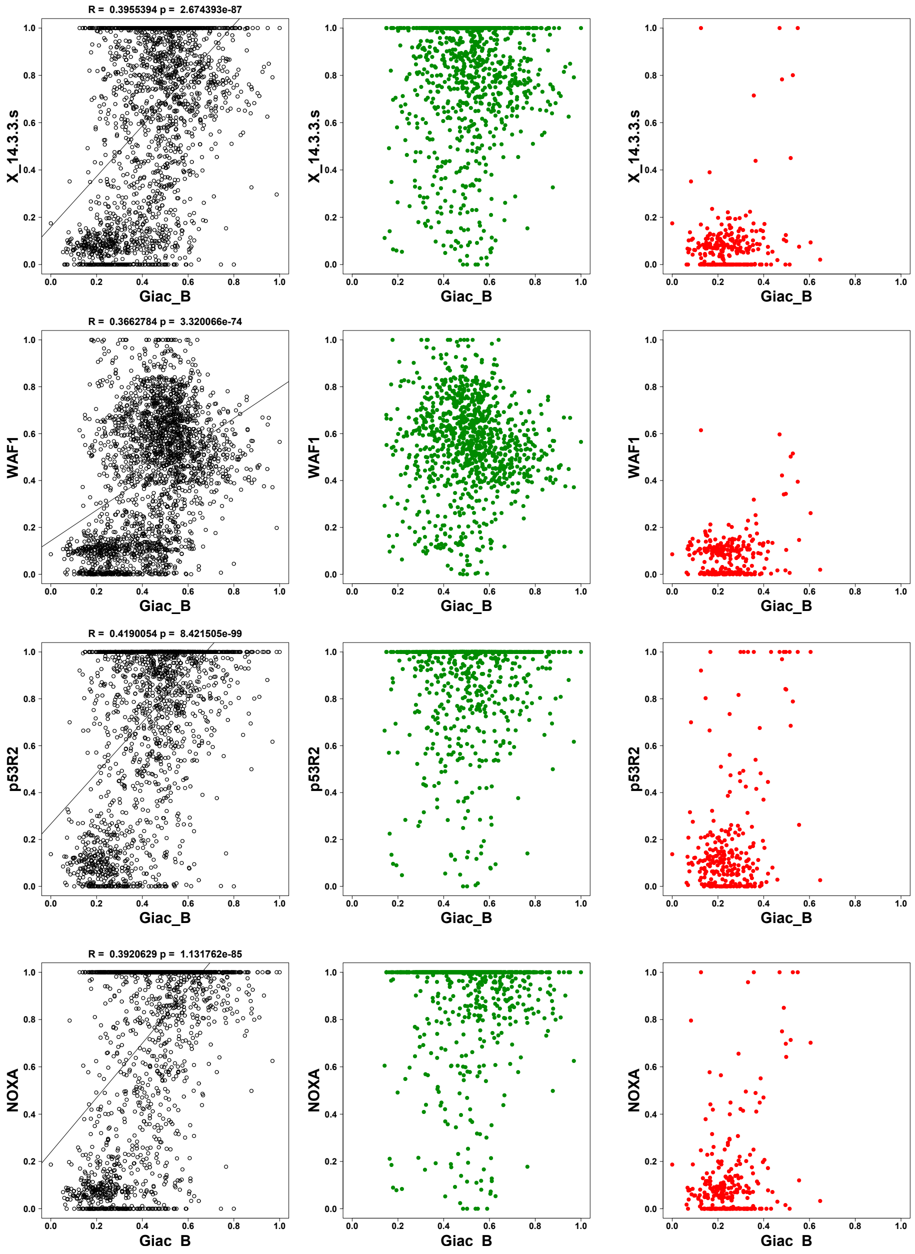

Supplementary Figure 3; panel 3C

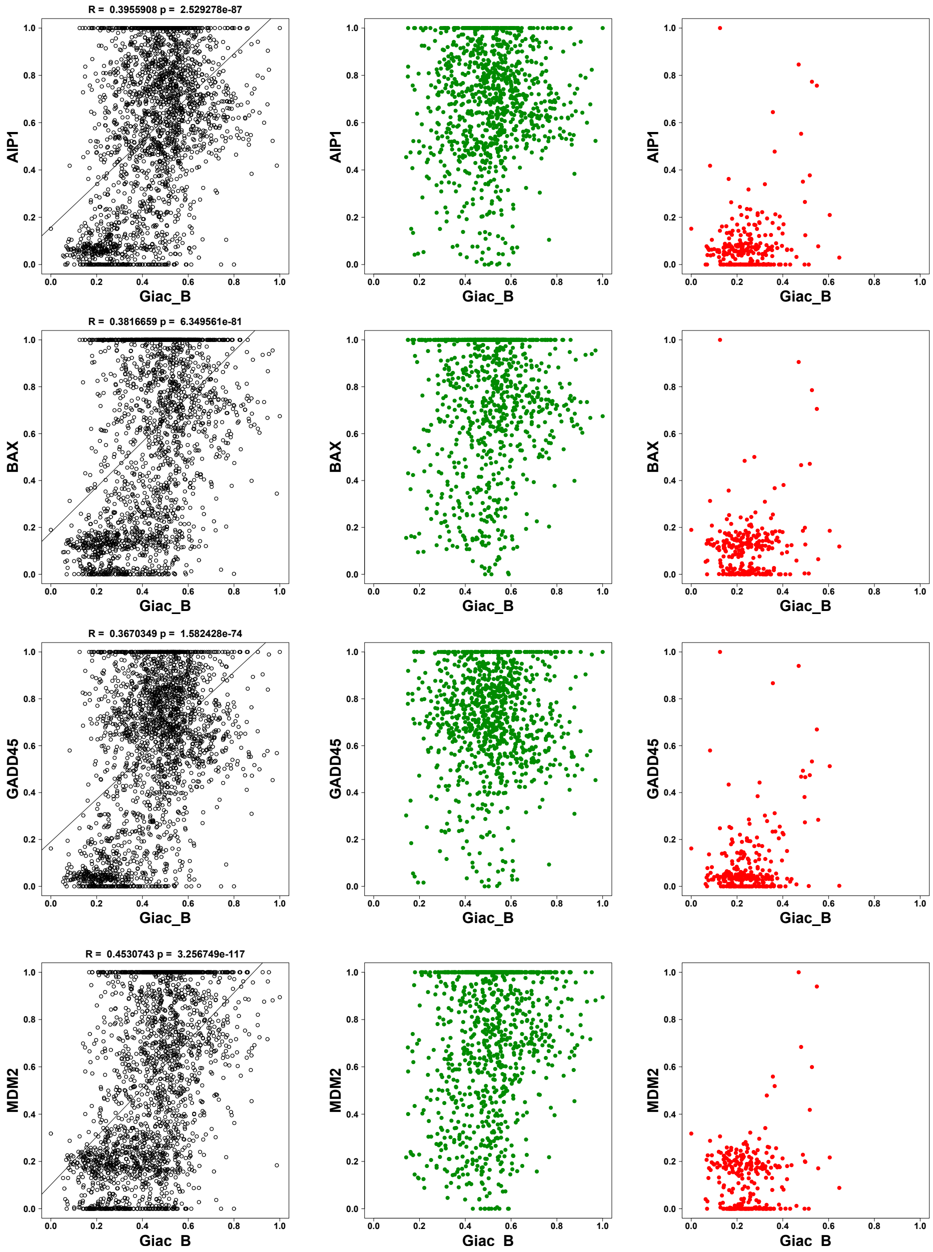

Supplementary Figure 3; panel 3C

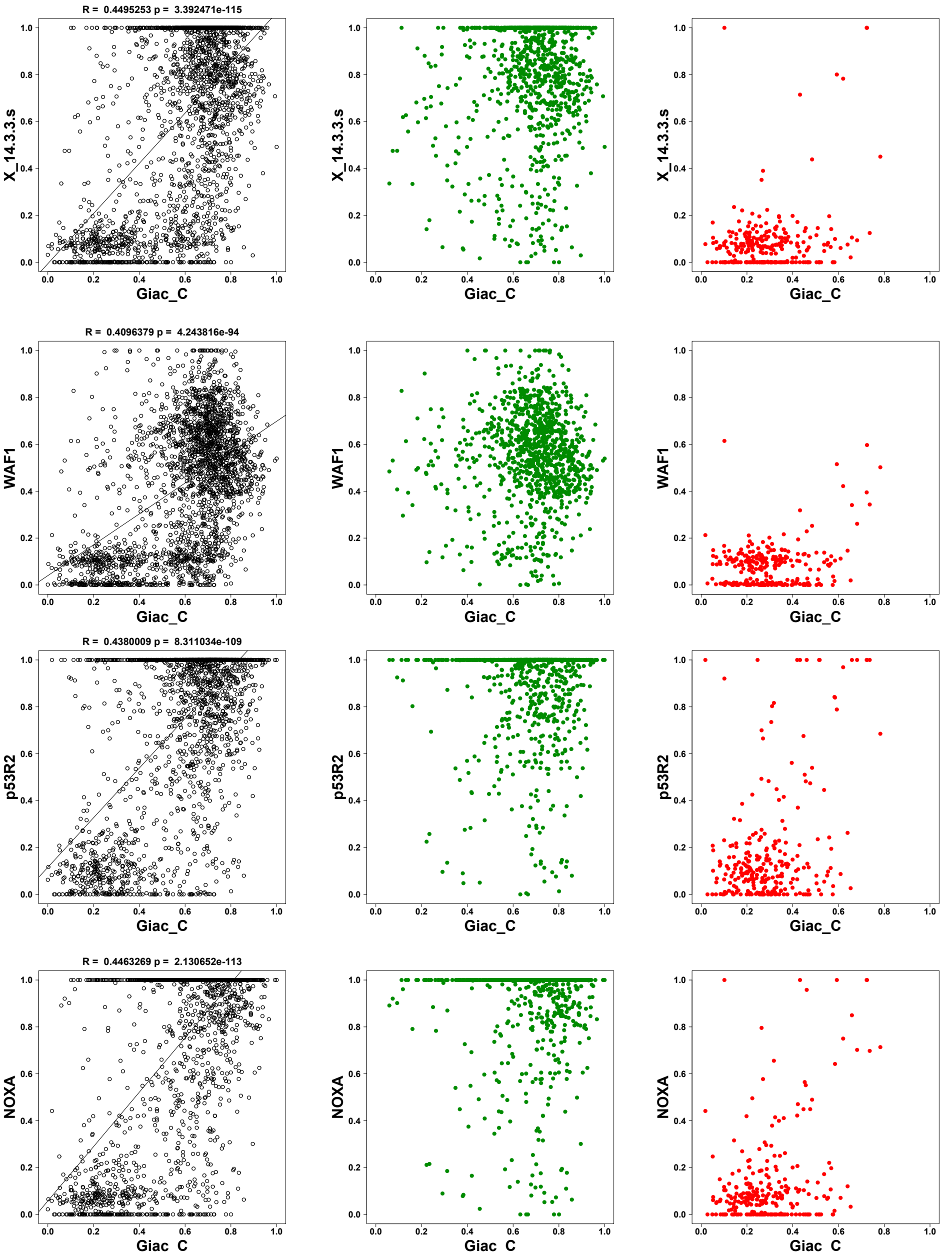

Supplementary Figure 3; panel 3D

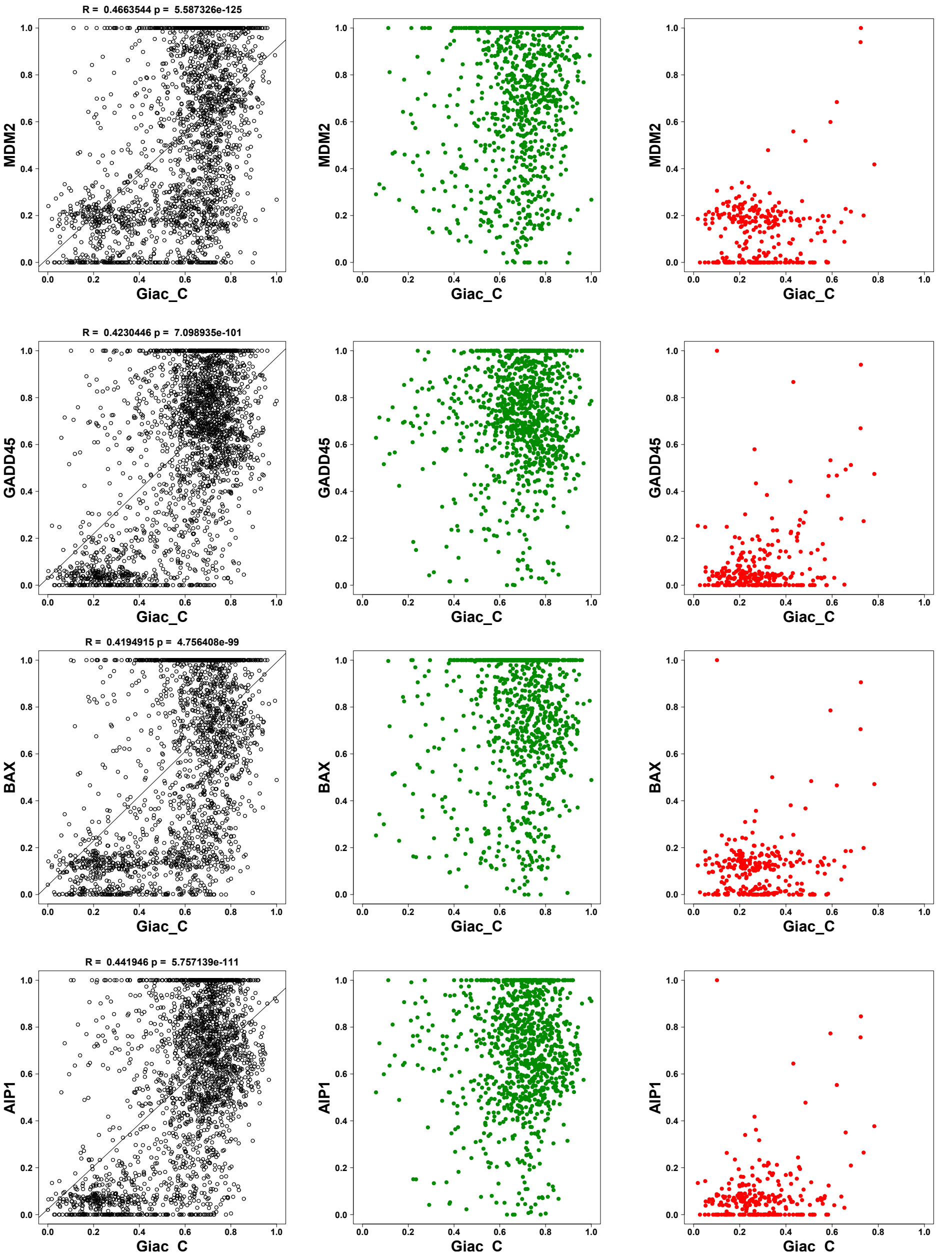

Supplementary Figure 3; panel 3D

Supplementary Figure 3: binary correlation analysis between the various read-out of TP53 loss of activity. Overall Pearson r-correlation coefficient and p value are shown at the top of each figure**.** The binary comparison between each analyzed study has between split in two panels according to the TP53 variants found in the CSD (red) or rare TP53 variant (green, variants found once or absent from the UMD database).

Panel 3A: binary comparison between the two studies performed in mammalian cells

Panels 3B, C and D: comparison between studies performed in yeast and in mammalian cells, Giac_A (3B), Giac_B (3C) and Giac_C (3D).

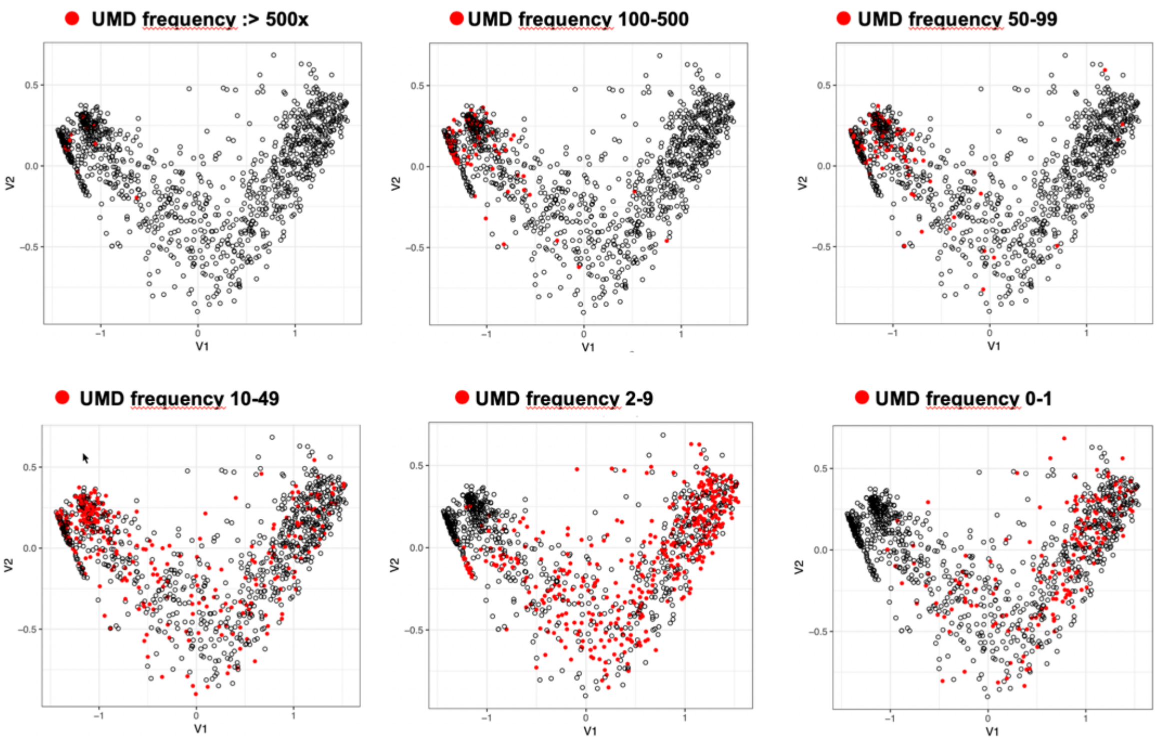

Supplementary Figure 4: Multidimensional scaling (MDS) for reduction of the 12 activity measures into bidimensional space. In each graph, the frequency of TP53 variant in the database are labeled in shown as red circles.

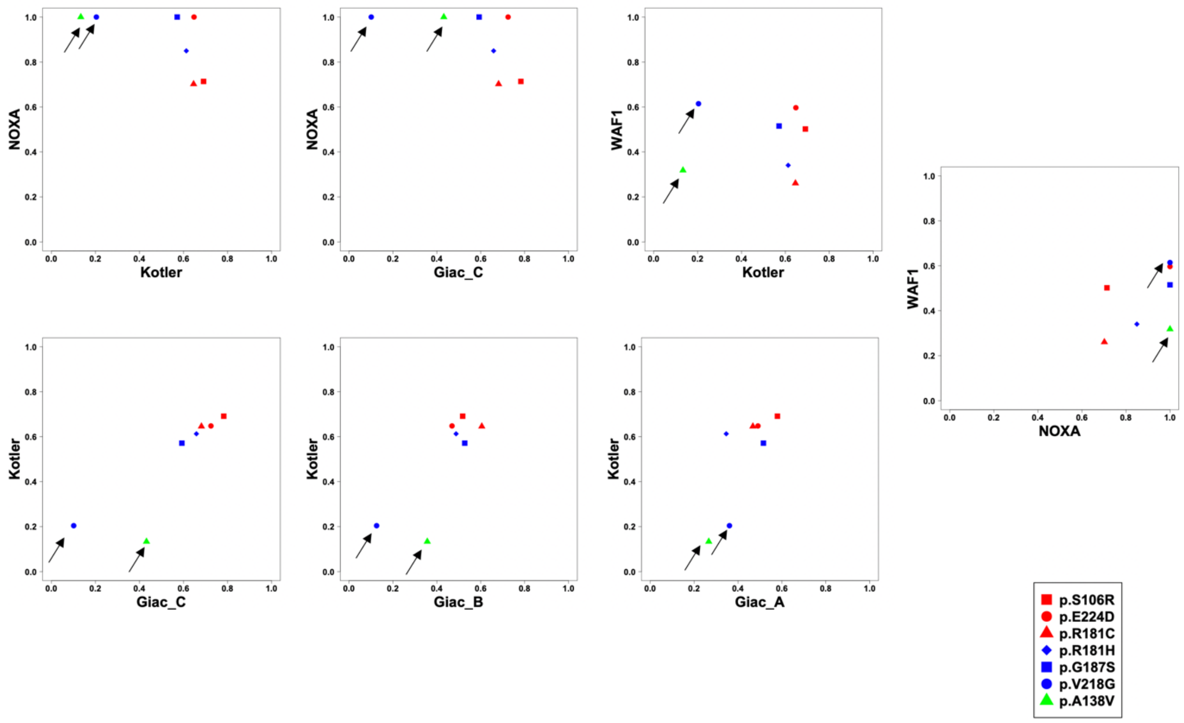

Supplementary Figure 5: Position of the 7 CSD outlier variants in different binary comparison. Two variants, p.A138V and p.218G (shown by black arrows), display no loss of activity in the yeast assay (top figures) but are inactive using the various mammalian readouts (bottom figures). Other variants associated with splice defects are also displayed in the figure.

▾ Tables

Potential splice sites | Potential Branch Points | Enhancer motifs | Silencer motifs | Other motifs

▾ ESE Finder matrices for SRp40, SC35, SF2/ASF and SRp55 proteins

Threshold values:
SF2/ASF: 72.98 SF2/ASF (IgM-BRCA1): 70.51 SRp40: 78.08 SC35: 75.05 SRp55: 73.86
Variation expresses the difference between reference and mutant values. Wild Type value is taken as reference.

Sequence Position	cDNA Position	Linked SR protein	Reference Motif (value 0-100)	Linked SR protein	Mutant Motif (value 0-100)	Variation
16	+16	SC35	ggcagcta (75.23)	SF2/ASF (IgM-BRCA1)	ggcaggt (74.31)	-1.23 %
16	+16	SC35	ggcagcta (75.23)	SF2/ASF	ggcaggt (82.59)	+9.78 %
21	+21	SF2/ASF (IgM-BRCA1)	ctacggt (74.38)			Site broken -100

▾ RESCUE ESE hexamers

No difference between mutant and reference sequence was found with this matrice.

▾ Predicted PESE Octamers from Zhang & Chasin

No Enhancer motif found with this matrice

▾ EIEs from Zhang et al.

Sequence Position	cDNA Position	Enhancer motif reference sequence	Enhancer motif mutant sequence	Variation
18	+18	cagcta		Site broken
19	+19	agctac		Site broken
20	+20	gctacg		Site broken
21	+21	ctacgg		Site broken

Supplementary Figure 6 : Variant c.318C>G (p.S106R) is predicted to alter TP53 splicing. Analysis of TP53 variant was performed with Human Splicing Finder (v3.1)(http://www.umd.be/HSF/).

Supplementary Video 1. 3D scatter plot of three different readouts of TP53 activity.
